# Single-cell RNA sequencing data locate ALDH1A2-mediated retinoic acid synthetic pathway to glomerular parietal epithelial cells

**DOI:** 10.3389/ebm.2024.10167

**Published:** 2024-09-18

**Authors:** Wen-Bin Liu, Damian Fermin, An-Long Xu, Jeffrey B. Kopp, Qihe Xu

**Affiliations:** ^1^ Beijing Research Institute of Chinese Medicine, Beijing University of Chinese Medicine, Beijing, China; ^2^ Department of Internal Medicine, Division of Nephrology, University of Michigan, Ann Arbor, MI, United States; ^3^ Kidney Disease Section, Kidney Diseases Branch, National Institute of Diabetes and Digestive and Kidney Diseases, National Institutes of Health, Bethesda, MD, United States; ^4^ Renal Sciences and Integrative Chinese Medicine Laboratory, Department of Inflammation Biology, School of Immunology & Microbial Sciences, Faculty of Life Sciences & Medicine, King’s College London, London, United Kingdom

**Keywords:** *Aldh1a2*, retinoic acid, scRNA-seq, snRNA-seq, acute kidney injury, chronic kidney disease, rapidly progressive glomerulonephritis, parietal epithelial cells

## Abstract

Aldehyde dehydrogenase 1, family member A2, is a retinoic acid-synthesizing enzyme encoded by *Aldh1a2* in mice and *ALDH1A2* in humans. This enzyme is indispensable for kidney development, but its role in kidney physiology and pathophysiology remains to be fully defined. In this review, we mined single-cell and single-nucleus RNA sequencing databases of mouse and human kidneys and found that glomerular parietal epithelial cells (PECs) express a full set of genes encoding proteins needed for cellular vitamin A uptake, intracellular transport, and metabolism into retinoic acid. In particular, *Aldh1a2/ALDH1A2* mRNAs are selectively enriched in mouse and human PECs. *Aldh1a2* expression in PECs is greatly increased in a mouse model of anti-glomerular basement membrane glomerulonephritis and moderately induced in a mouse model of ischemia-reperfusion acute kidney injury. *Aldh1a2* expression in PECs is substantially repressed in a chronic kidney disease mouse model combining diabetes, hypertension, and partial nephrectomy and is moderately repressed in mouse models of focal segmental glomerulosclerosis and diabetic nephropathy. Single-nucleus RNA sequencing data show that *ALDH1A2* mRNA expression in PECs is diminished in patients with chronic kidney disease associated with diabetes, hypertension and polycystic kidney disease. In addition to data mining, we also performed Spearman’s rank correlation coefficient analyses and identified gene transcripts correlated with *Aldh1a2/ALDH1A2* transcripts in mouse PECs and PEC subtypes, and in human PECs of healthy subjects and patients with AKI or CKD. Furthermore, we conducted Gene Ontology pathway analyses and identified the biological pathways enriched among these *Aldh1a2/ALDH1A2*-correlated genes. Our data mining and analyses led us to hypothesize that ALDH1A2*-*mediated retinoic acid synthesis in PECs plays a yet-undefined role in the kidney and that its dysregulation mediates injury. Conditional, PEC-selective *Aldh1a2* knockout, RNA silencing and transgenic mouse models will be useful tools to test this hypothesis. Clinical studies on genetics, epigenetics, expression and functions of *ALDH1A2* and other genes needed for retinoic acid biosynthesis and signaling are also warranted.

## Impact statement

In mice, the *Aldh1a2* gene is indispensable for kidney development. In humans, loss-of-function mutations of *ALDH1A2* are not tolerated and partial loss-of-function mutations lead to severe developmental problems in multiple organs, including the kidney. However, the exact kidney cell types that express *Aldh1a2/ALDH1A2* in adult kidneys and the roles of retinoic acid signalling in these cells are poorly understood. By mining the latest scRNA-seq and snRNA-seq databases of mouse and human kidneys in health and disease, we highlight glomerular PECs as the major site of *Aldh1a2/ALDH1A2* expression and this expression is differentially dysregulated in different kidney diseases. We hypothesize that normal *ALDH1A2*-mediated retinoic acid synthesis in PECs plays important roles in maintaining kidney health and defending against disease. Further experimental and clinical studies against this hypothesis may lead to novel strategies for stratified diagnosis, cost-effective prevention, and efficacious treatment of kidney diseases.

## Introduction

**FIGURE 1:**
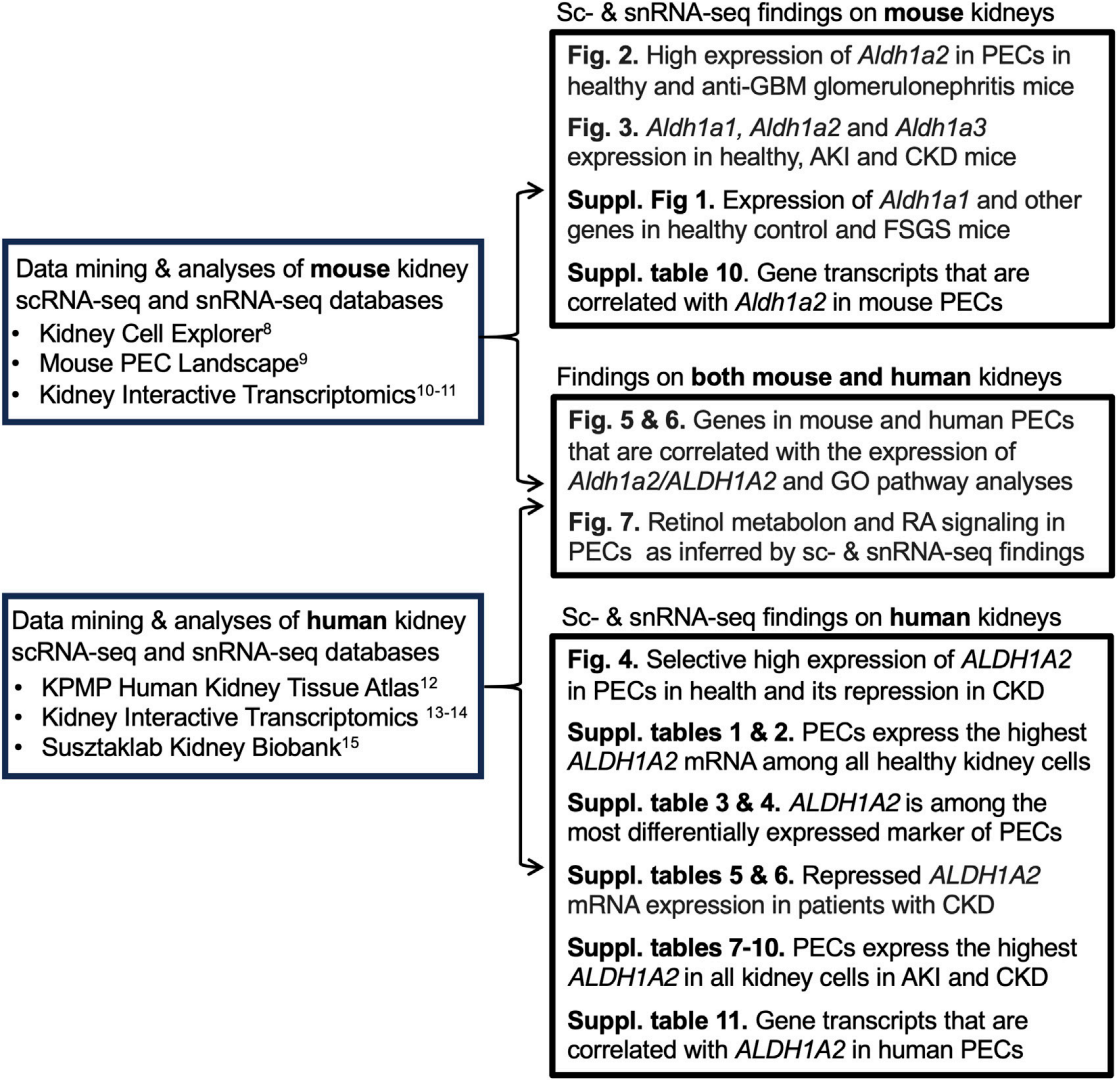
Analytical workflow of our data analyses and findings.

Aldehyde dehydrogenase 1 family member A2 (Aldh1a2/ALDH1A2) is an enzyme that catalyzes the second-step oxidation of vitamin A (retinol) and the irreversible conversion of retinaldehyde to retinoic acid (RA). In mice, the enzyme is encoded by *Aldh1a2*, which is indispensable for kidney development [1, 2]. In humans, the enzyme is encoded by *ALDH1A2*, an intronic variant of which causes increased enzyme activity and is associated with increased serum RA and newborn kidney size [3]. On the other hand, biallelic partially loss-of-function *ALDH1A2* coding variants result in severe congenital anomaly syndromes, including small kidneys and neonatal lethality [4, 5]. In addition, non-coding *ALDH1A2* variants reduce *ALDH1A2* expression in chondrocytes and contribute to severe hand osteoarthritis in adults [6]. This is in keeping with the notion that RA biosynthesis may continue to be active in post-natal life and may play cell-type specific roles [7]. However, whether *Aldh1a2* and *ALDH1A2* play any roles in kidney health and disease remains to be fully characterized. To address this issue, we mined single-cell RNA sequencing (scRNA-seq) and single-nucleus RNA sequencing (snRNA-seq) databases (Table 1) to evaluate *Aldh1a2/ALDH1A2* expression in different kidney cell types in mice and humans and examine how expression changes in kidney diseases. It is hoped that these data will facilitate generating hypotheses on the role of *Aldh1a2* and *ALDH1A2* in kidney health and disease and will guide in devising strategies for further studies. Our analytical workflow is illustrated in Figure 1.

**TABLE 1:** Databases explored in the present study.

Database category and name	Website	Date of last access
*Mouse databases*		
Kidney Cell Explorer [8]	https://cello.shinyapps.io/kidneycellexplorer/	6th Feb. 2024
Mouse PEC Landscape [9]	https://wenbinliu.shinyapps.io/mouse_PECs/	6th Feb. 2024
Kidney Interactive Transcriptomics 1 million cell atlas of mouse DKD and its treatments [10] Mouse IRI Kidney [11]	http://humphreyslab.com/SingleCell	6th Feb. 2024 6th Feb. 2024
*Human databases*		
KPMP Human Kidney Tissue Atlas [12]	https://atlas.kpmp.org/explorer/dataviz	6th Feb. 2024
Kidney Interactive Transcriptomics Human DKD snRNA + scATAC-seq [13] Human ADPKD snRNA + scATAC-seq [14]	http://humphreyslab.com/SingleCell	6th Feb. 2024 6th Feb. 2024
Susztaklab Kidney Biobank [15]	https://susztaklab.com/hk_genemap/snRNA	6th Feb. 2024
Human Protein Atlas [16]	https://www.proteinatlas.org/ENSG00000128918-ALDH1A2/tissue/kidney#	6th Feb. 2024

## Data from mouse models

**FIGURE 2:**
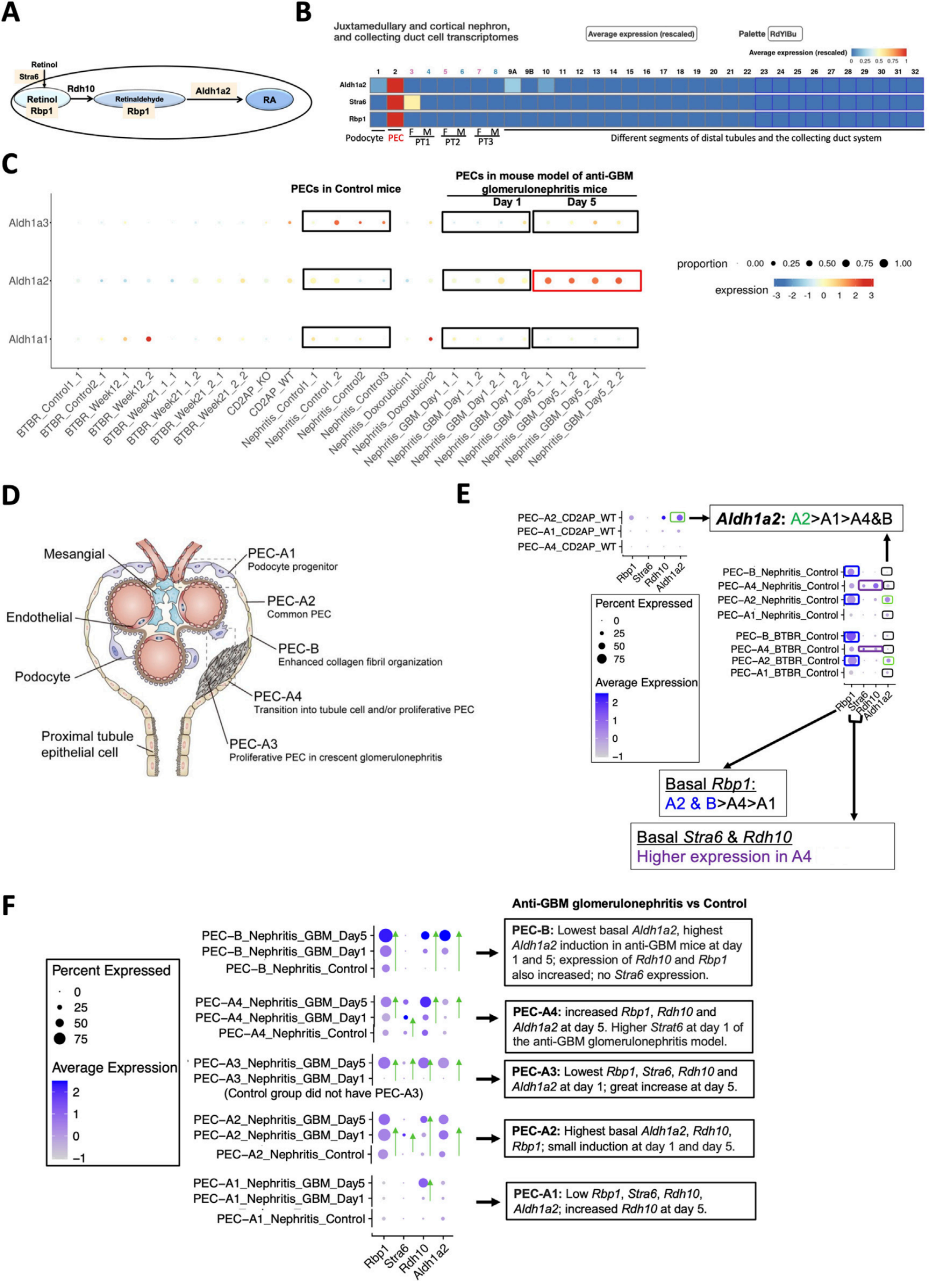
Mouse PECs have high *Stra6*, *Rbp1* and *Aldh1a2* mRNA expression, which is further induced in anti-GBM glomerulonephritis mice. **(A)** Diagram illustrates roles of Aldh1a2, Stra6, Rbp1 and Rdh10 proteins in the retinol metabolon. RA: retinoic acid. Pink shades indicate selective *Aldh1a2*, *Stra6* and *Rbp1* mRNA expression in mouse PECs. **(B)** Mouse renal scRNA-seq data were extracted from the Kidney Cell Explorer website. Relative average expression levels are color-coded (red > yellow > blue) with the highest expression normalized as 1. PT1, PT2 and PT3 denote segments 1, 2 and 3 of proximal tubule; F: female; M: male. **(C)** ScRNA-seq analysis of mouse PECs revealed distinct changes in mRNA expression of *Aldh1a1*, *Aldh1a2* and *Aldh1a3* in anti-GBM glomerulonephritis mice compared to healthy controls. Dot sizes indicate the relative proportion of PECs expressing the specific *Aldh1a* isoform, while the relative expression levels are color-coded, shown on a spectrum of blue (low expression) to red (high expression). To facilitate comparison, control and anti-GBM glomerulonephritis mice from day-1 and day-5 samples, among others, are highlighted by rectangle selection and text description. Nephritis_Control: samples of the control mice; Nephritis_GBM: samples of the anti-GBM glomerulonephritis mice. Other groups are irrelevant to studies of the anti-GBM glomerulonephritis mice but are nonetheless shown to demonstrate the specificity of the changes observed in the anti-GBM glomerulonephritis mice. **(D)** Diagram illustrates different PEC subtypes, among other glomerular cell types, adapted from Liu et al 2023 [9]. **(E)** ScRNA-seq analysis of PEC subtypes in wild-type and healthy control mice revealed differing levels of physiological mRNA expression of *Aldh1a2*, *Stra6, Rbp1 and Rdh10*. The bubble plot shows expression levels of *Aldh1a2*, *Stra6, Rbp1* and *Rdh10* in different PEC subtypes. Dot sizes indicate the proportion of PECs expressing a specific gene and brighter blue color indicates higher expression levels. The highest expression of *Aldh1a2* in PEC-A2 is highlighted by green rectangle selection; higher expression of *Rbp1* in PEC-A2 and PEC-B is highlighted by blue rectangle selection, while higher expression of *Stra6* and *Rdh10* in PEC-A4 is indicated by purple rectangle selection. **(F)** ScRNA-seq analysis of healthy control versus anti-GBM glomerulonephritis mice (day 1 and 5) revealed different changes in mRNA expression of *Aldh1a2*, *Stra6, Rbp1* and *Rdh10* in PEC subtypes. Dot sizes indicate the proportion of PECs expressing a specific gene; brighter blue color indicates higher expression levels. The trend of increase in expression is indicated by green arrows pointing to the higher expression. Cd2ap_WT are wild-type control mice for comparison with *Cd2ap* knockout mice (Supplementary Figure S1); Nephritis_Control: control mice RNA expression; Nephritis_GBM: anti-GBM glomerulonephritis mouse RNA expression.

While mining the scRNA-seq database, Mouse Kidney Cell Explorer (Table 1), we discovered that healthy mouse PECs expressed *Stra6*, *Rbp1* and *Aldh1a2*, in a selective manner compared to other kidney cell types and did so at relatively high levels. These genes respectively encode a retinol uptake receptor, an intracellular retinol-binding protein and an enzyme catalyzing retinaldehyde conversion to retinoic acid (RA) (Figures 2A, B) [8]. Mouse PECs also manifested abundant expression of the ubiquitously expressed *Rdh10*, which encodes the main enzyme converting retinol to retinaldehyde. Hence, mouse PECs are likely active in RA synthesis.

As Aldh1a2/ALDH1A2 is the main enzyme catalyzing the final step of RA biosynthesis, we are particularly interested its expression and function. We hypothesize that Aldh1a2/ALDH1A2 in PECs, through catalyzing RA biosynthesis, play important roles in kidney health, and that their dysregulation contributes to kidney disease. To address this hypothesis, we gathered experimental evidence from published mouse models of acute kidney injury (AKI) and chronic kidney disease (CKD), particularly those manifesting injury to, or activation of, PECs and/or podocytes, *e.g.,* anti-glomerular basement membrane (GBM) glomerulonephritis, which often manifests as rapidly progressive glomerulonephritis (RPGN); primary podocyte diseases minimal change disease (MCD) and focal segmental glomerulosclerosis (FSGS), which often manifest as nephrotic syndrome. We also examined the data for mouse models of diabetic nephropathy, which may manifest podocyte injury and is the most common CKD in the developed world.

Analysis of the Mouse PEC Landscape database containing scRNA-seq gene expression data for PECs from healthy mice and mouse models of kidney disease [9] (Table 1) revealed that *Aldh1a2* expression in PECs increased substantially in murine anti-GBM glomerulonephritis, on day 5 after intravenous injection of sheep anti-rat glomeruli serum (Figure 2C). In contrast, the expression of *Aldh1a3* decreased, while no change in *Aldh1a1* expression was observed in this model. Hence, Aldh1a2 likely plays the main role in PECs in this anti-GBM glomerulonephritis mouse model, although Aldh1a1, -2 and -3 isoenzymes all accomplish the final step of RA biosynthesis. Given that PECs play major roles in anti-GBM glomerulonephritis [17], it is compelling to further examine the expression and role of *Aldh1a2* in PECs in health and in anti-GBM glomerulonephritis.

As shown in Figure 2D, mouse PECs can be subdivided into five sub-types: PEC-A1 (podocyte progenitor), PEC-A2 (common PEC), PEC-A3 (proliferative PEC contributing to crescent formation), PEC-A4 (tubular progenitor) and PEC-B (pro-fibrogenic PECs). PEC-A1, A2, A4 and PEC-B are present in both healthy and diseased kidneys, while PEC-A3 only have been found in murine anti-GBM glomerulonephritis [9]. We compared *Aldh1a2, Stra6*, *Rbp1* and *Rdh10* mRNA expression in these PEC subtypes. As shown in Figure 2E, PEC subtypes differ in basal expression of *Aldh1a2*: the highest *Aldh1a2* expression was observed in PEC-A2 in wild-type healthy control mice. Wild-type healthy control mouse PEC-A2 also have higher expression of *Rbp1* compared with other PEC sub-types. In contrast, wild-type healthy mouse PEC-B had the lowest expression of *Aldh1a2*. As shown in Figure 2F, in anti-GBM glomerulonephritis mice, *Aldh1a2* expression was progressively and greatly induced in PEC-B, mildly increased in PEC-A2, and was substantially higher on day 5 than on day 1 in PEC-A3 cells, but expression did not change in PEC-A1 cells. Consequently, by day 5, PEC-B cells became the PEC subtype with the highest expression of *Aldh1a2*. PEC-B cells are a minor PEC subtype in healthy mice but numbers increase progressively and become the major PEC subtype in anti-GBM glomerulonephritis mice by day 5 [9]. It will be important to determine the role of *Aldh1a2* in PEC-B in anti-GBM glomerulonephritis. Supporting the concept that increased *Aldh1a2* expression in PEC-B plays particular roles by catalyzing retinol activation and RA synthesis, the substantial progressive induction of *Aldh1a2* expression in PEC-B paralleled the similar, progressive induction of *Rbp1* and *Rdh10* expression in PEC-B (Figure 2F).

PEC-A2 not only had the highest basal expression of *Aldh1a2* and *Rbp1* but also manifested relatively high expression in mouse anti-GBM glomerulonephritis on days 1 and 5, suggesting that RA signaling in PEC-A2 may also contribute to pathogenesis. In PEC-B, PEC-A3 and PEC-A4, *Aldh1a2*, *Rbp1* and *Rdh10* expression increased on day 5 versus day 1. This suggests that RA signaling in these PEC subtypes may play particular roles in the later stage of anti-GBM glomerulonephritis. In contrast, PEC-A1 had little *Aldh1a2, Rdh10, Stra6* and *Rbp1* expression in control mice and on day 1 of anti-GBM glomerulonephritis but had higher *Rdh10* mRNA expression on day-5 compared to day-1 in anti-GBM glomerulonephritis (Figure 2F).

PECs have intimate crosstalk with podocytes and may contribute to FSGS pathogenesis [18]. Towards understanding whether RA signaling might play a role in experimental FSGS, we compared *Aldh1a2, Stra6*, *Rbp1* and *Rdh10* mRNA expression in different PEC subtypes in FSGS mouse models, induced either by doxorubicin or by *Cd2ap* gene knockout, compared to healthy control mice. As shown in Supplementary Figure S1, expression of *Aldh1a2, Stra6*, *Rbp1* and *Rdh10* in PEC sub-types was not induced in mouse models of FSGS and, instead, showed a trend toward repression in some PEC subtypes in the disease models.

**FIGURE 3:**
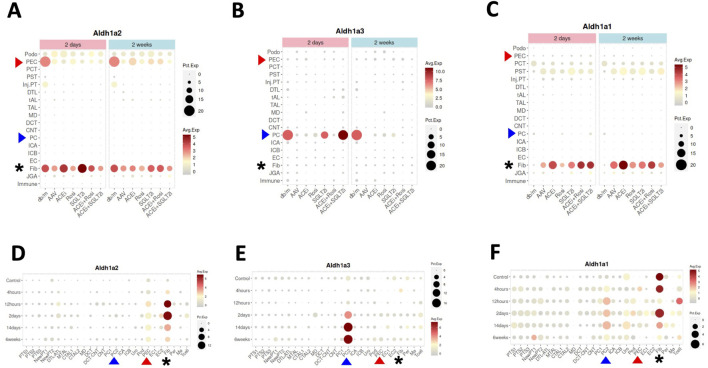
*Aldh1a1, Aldh1a2* and *Aldh1a3* expression in kidney cells of female healthy and CKD mice, and of male healthy and AKI mice induced by ischemia-reperfusion. **(A–C)** show *Aldh1a2*, *Aldh1a3* and *Aldh1a1* mRNA expression in the CKD study and **(D–F)** show *Aldh1a2*, *Aldh1a3* and *Aldh1a1* mRNA expression in the AKI study. Dot sizes indicate the proportion of cells expressing a particular gene. Expression levels are color-coded. Red and blue arrowheads point to gene expression in PECs and PCs, respectively, while the expression levels of fibroblasts are marked by black asterisks. As shown in **(A–C)**, neither repression of *Aldh1a2* expression in PECs and *Aldh1a3* expression in medullary PCs (PC2), nor induction of *Aldh1a1* expression in fibroblasts in the AAV mice (a CKD model characterized by hypertension induced by unilateral nephrectomy and adeno-associated virus mediated renin transgene in *db/db* mice) versus in that of healthy controls (db/m) was effectively rescued by various treatment regimens, especially at the 2-week time point. ACEi: CKD treated with angiotensinogen converting enzyme (ACE) inhibitor; Rosi: CKD treated with PPARγ pharmacological agonist rosiglitazone; SGLT2i: CKD treated with sodium-glucose cotransporter-2 inhibitor; ACEi + Rosi: CKD treated with ACE inhibitor and the thiazolidinedione rosiglitazone; ACEi + SGLT2i: CKD treated with an ACE inhibitor and an SGLT2 inhibitor. As shown in **(D–F)**, in the setting of AKI, *Aldh1a2* expression in PECs and fibroblasts, *Aldh1a3* expression in PC2, and A*ldh1a1* expression in PC2 were all induced, while *Aldh1a1* expression in fibroblasts was repressed in a biphasic fashion. The Y-axis presents data from control mice and experimental group mice from 4 h to 6 weeks after ischemia-reperfusion injury. Data were downloaded with permission from Kidney Interactive Transcriptomics [10, 11].

Next, we searched the Kidney Interactive Transcriptomics database (Table 1), particularly focusing on data from a recent scRNA-seq study of a multifactorial CKD model in female mice in comparison with female wild-type control mice — the CKD model (AAV mice) was characterized by obesity and diabetes caused by a homozygous point mutation in the gene for the leptin receptor (*db/db*), hypertension induced by adeno-associated virus mediated renin transgene combined with unilateral nephrectomy [10]. In healthy control mice (db/m), selective high expression of *Aldh1a2*, and low level expression of *Aldh1a1* and *Aldh1a3*, were found in PECs (Figures 3A–C). *Aldh1a2* expression in PECs and *Aldh1a3* expression in the collecting duct principal cells (PCs) were markedly repressed in the CKD model (AAV mice). Neither was effectively rescued by a peroxisome proliferator-activated receptor (PPAR)-γ agonist, an angiotensinogen converting enzyme inhibitor, a sodium-glucose cotransporter-2 inhibitor, or combined therapies (Figures 3A, B) [10].

Because *Aldh1a2* and *Aldh1a3* are the major RA synthesizing enzymes in PECs and PCs, respectively, repressed expression could cause reduced RA activity in these cells. Exposure of cultured PCs to media containing aldosterone, angiotensin II or high glucose repressed retinoic acid receptor (RAR)-dependent RA signaling [19]. Thus, repression of *Aldh1a3* expression in PCs by these (and other) CKD mediators is a plausible mechanism for the repressed RA/RAR signaling pathway in PCs in this complex, but clinically relevant, CKD murine model.

As our previous work has shown that RA/RAR activities in PCs show opposite responses to acute versus chronic kidney injury stimuli [19], we asked whether *Aldh1a2* expression in PECs might also change differentially in AKI versus CKD. By further exploring the Kidney Interactive Transcriptomics database, we found that *Aldh1a2* in PECs, and *Aldh1a1* and *Aldh1a3* expression in medullary PCs (PC2) were induced in male mice subjected to ischemia-reperfusion AKI. Further, *Aldh1a1* expression in male fibroblasts was repressed at 12 h after ischemia-reperfusion, had recovered at 2 days, and was again repressed at 2–6 weeks (Figures 3D–F) [11]. These were in contrast with findings in female CKD mice, in which *Aldh1a2* expression in PECs and *Aldh1a3* expression in PCs were both repressed (Figures 3A, B). It deserves further exploration whether these differences are due to opposite effects of AKI and CKD, or due to other factors, e.g., sex.

## Data from human studies

**FIGURE 4:**
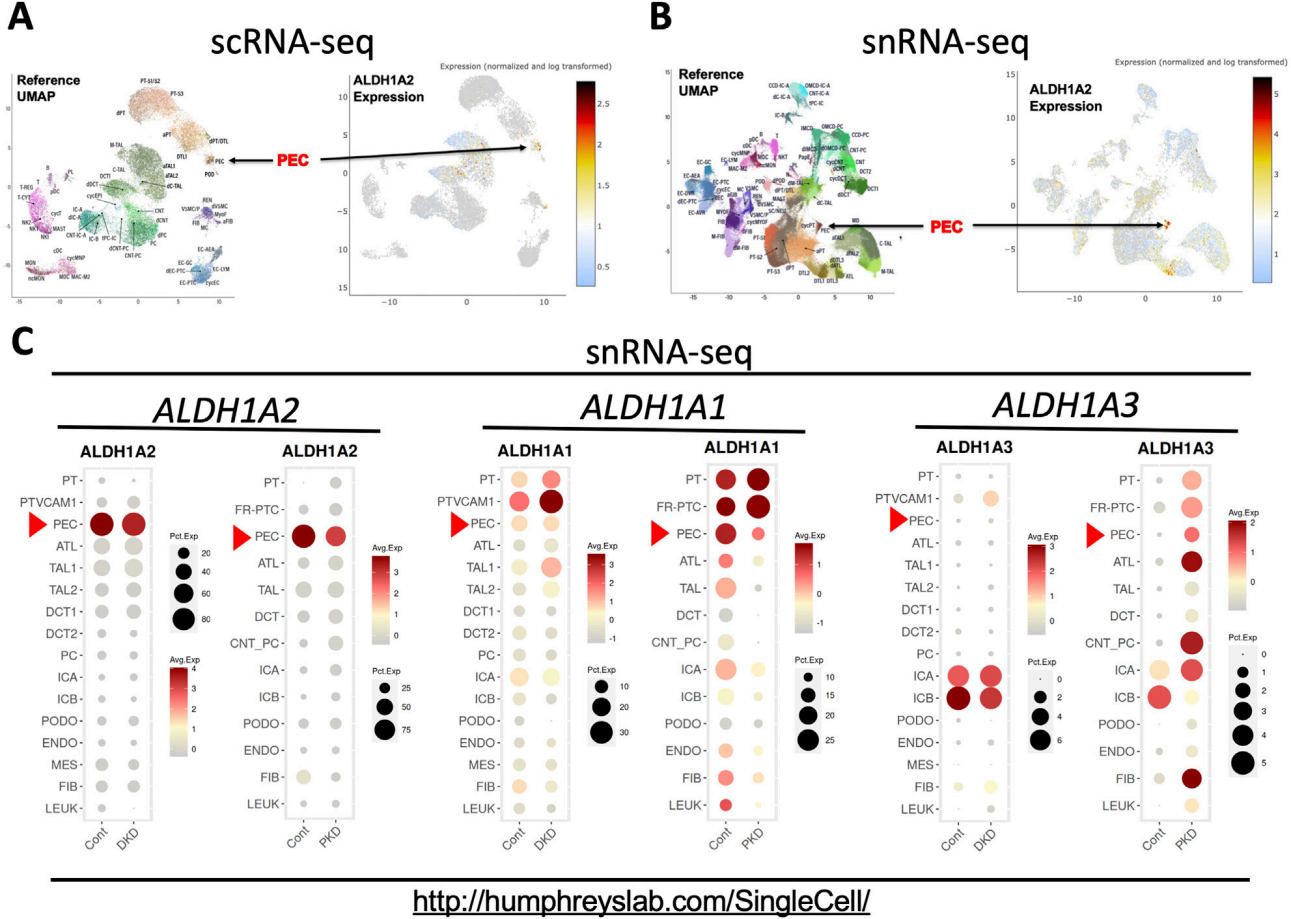
scRNA-seq and snRNA-seq analysis shows that selective high expression of *ALDH1A2* expression in PECs in healthy subjects is repressed in patients with CKD. **(A,B)** Shown are the reference uniform manifold approximation and projection (UMAP) data, the UMAP illustration of *ALDH1A2* expression of the scRNA-seq, and the snRNA-seq data, respectively. The names and positions of particular cell types are shown as distinct clusters in the reference UMAPs (left). The normalized and log transformed expression of *ALDH1A2* mRNA in these clusters are color-coded and shown in the right panels. Arrows point to the PEC clusters. The data are downloaded from the Human Kidney Tissue Atlas (https://atlas.kpmp.org/explorer/dataviz) of the Kidney Precision Medicine Project with permission [12]. Accessed on 6th February 2024. Funded by the National Institute of Diabetes and Digestive and Kidney Diseases (Grant numbers are listed in the table key to Supplementary Table S1). **(C)** shows snRNA-seq analysis of *ALDH1A1*, *ALDH1A2 and ALDH1A3* expression in healthy subjects and patients with diabetic kidney disease (DKD) and late-stage ADPKD (PKD). Dot sizes indicate the proportion of cells expressing a particular gene; relative gene expression levels are color-coded. The web link shows the source of the displayed information. Red arrowheads point to the mRNA expression in PECs. Data were downloaded with permission from Kidney Interactive Transcriptomics [13, 14].

The Kidney Precision Medicine Project (KPMP), part of the Human BioMolecular Atlas Program [20], has recently published the online Human Kidney Tissue Atlas (Table 1), containing scRNA-seq and snRNA-seq analyses of kidney tissues from healthy subjects and patients with AKI and CKD [12]. Both scRNA-seq and snRNA-seq indicated that PECs had the highest expression of *ALDH1A2* mRNA among all kidney cell types (Figures 4A, B). In the scRNA-seq dataset, only PECs, thick ascending limb (TAL) cells and degenerated proximal tubule epithelial cells and descending thin limb (DTL) cells differentially expressed *ALDH1A2* mRNA, with PECs having the highest cellular expression percentage (35%) and the highest mean expression level (Supplementary Table S1). In the snRNA-seq dataset, *ALDH1A2* was differentially expressed in PECs and a few other cell types, with PECs having the highest percentage (88%) and mean expression level (Supplementary Table S2). In the scRNA-seq and snRNA-seq datasets, *ALDH1A2* was the No. 129 (Supplementary Table S3) and No. 1 (Supplementary Table S4) most differentially expressed gene in PECs, respectively.

Further supporting highly cell-specific and abundant expression of *ALDH1A2* mRNA in healthy human PECs, the Kidney Interactive Transcriptomics snRNA-seq datasets from the Humphrey Lab and the snRNA-seq dataset of the Susztak Lab (Table 1) both reported that over 80% PECs had positive expression of *ALDH1A2* mRNA (Figure 4C; Supplementary Table S5) [13–15]. The selective, high physiological *ALDH1A2* mRNA expression in human PECs contrasts with the low physiological *ALDH1A3* expression in all renal cells, including PECs, and the ubiquitous expression *of ALDH1A1* in most renal cells and modest expression in PECs (Figure 4C). Hence, both scRNA-seq and snRNA-seq studies support *ALDH1A2* as selectively expressed at the mRNA level in healthy human PECs.

Next, we queried the KPMP datasets for *ALDH1A2* expression in healthy subjects (Supplementary Tables S1, S2) and CKD patients (Supplementary Tables S6, S7). A moderate reduction in *ALDH1A2*-expressing percentages in PECs was found in CKD patients compared to healthy controls, both in scRNA-seq (27% vs. 35%) and snRNA-seq datasets (77% vs. 88%). In subjects with AKI, however, this trend was observed in the scRNA-seq (25%), but not the snRNA-seq dataset (89%) (Supplementary Tables S8, S9).

To further examine how *ALDH1A2* mRNA expression in PECs changes in CKD patients, we assessed the Kidney Interactive Transcriptomics snRNA-seq analysis of patients with mild to moderate diabetic nephropathy and end-stage autosomal dominant polycystic kidney disease (ADPKD), and the Susztak Lab snRNA-seq analysis of kidney tissues from patients with CKD associated with diabetes or hypertension (Table 1) [13–15]. A reduction in *ALDH1A2* mRNA expression in PECs was observed in all these CKD subject cohorts, compared with healthy controls (Figure 4C; Supplementary Table S5).

## Integrated analysis of *Aldh1a2/ALDH1A2* and other genes in mouse and human PECs

**FIGURE 5:**
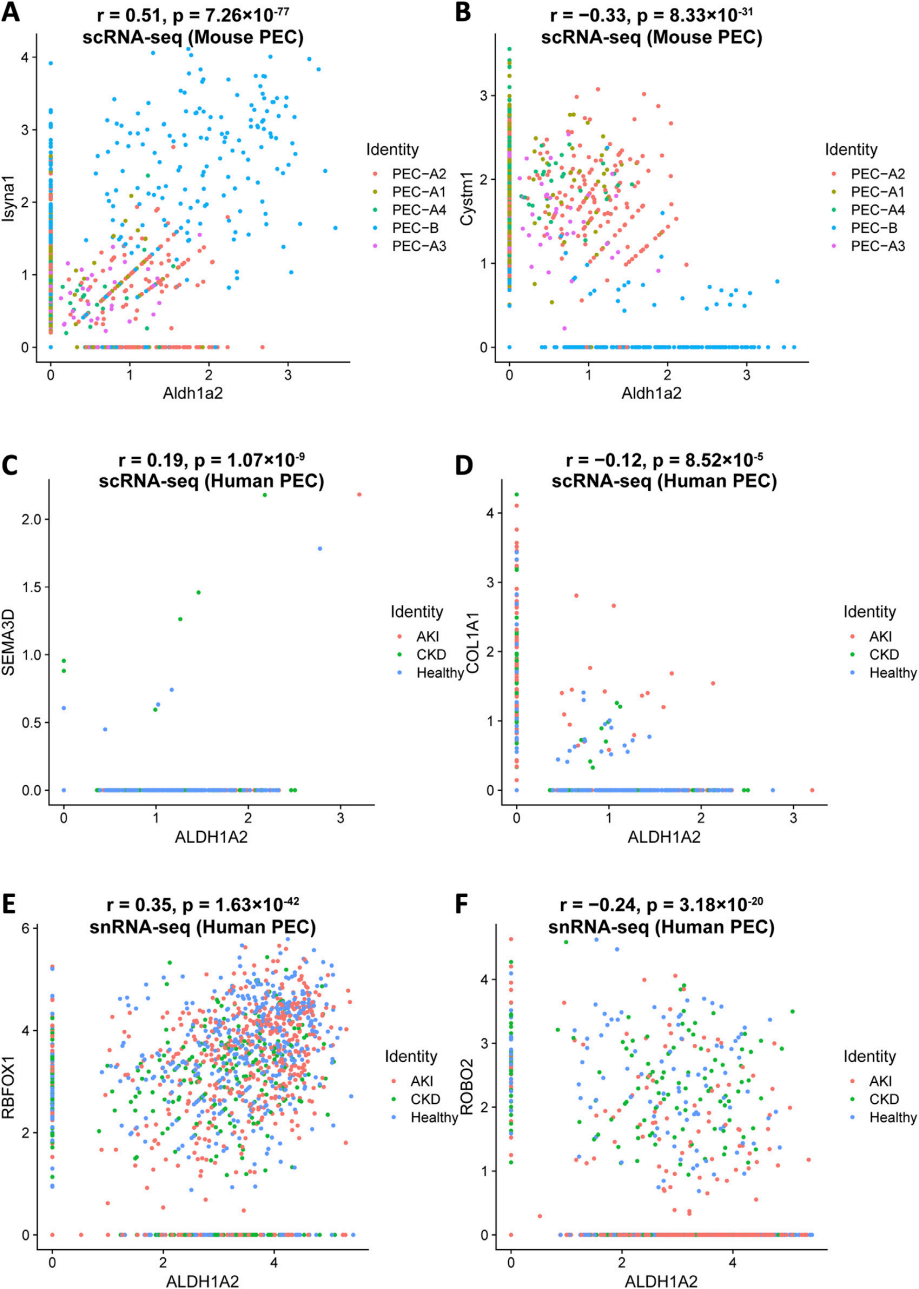
Top genes most significantly correlated with *Aldh1a2/ALDH1A2* expression in mouse and human PECs. Illustrations of top gene transcripts most significantly correlated with the *Aldh1a2* transcript in all mouse PEC subtypes in the Mouse PEC Landscape dataset (Supplementary Tables S10.1 and 10.2) and gene transcripts most significantly correlated with the *ALDH1A2* transcript in all human PECs in both scRNA-seq and snRNA-seq datasets of the KPMP Human Kidney Tissue Atlas (Supplementary Tables S11.1–11.4). **(A,B)** ScRNA-seq scatter plots showing the gene transcripts that are the most positively **(A)** and negatively **(B)** correlated with the transcript abundance of *Aldh1a2* in mouse PECs, respectively. **(C,D)** ScRNA-seq scatter plots showing the gene transcripts that are the most positively **(C)** and negatively **(D)** correlated with the transcript abundance of *ALDH1A2* in human PECs, respectively. **(E,F)** SnRNA-seq scatter plots showing the gene transcripts that are the most positively **(E)** and negatively **(F)** correlated with the transcript abundance of *ALDH1A2* in human PECs, respectively.

Understanding how *Aldh1a2/ALDH1A2* expression correlates with that of other genes in PECs might direct further causal studies to identify up- and downstream genes of *Aldh1a2/ALDH1A2* and other genes that are co-regulated for physiological or pathophysiological reasons. To this end, we analyzed the Mouse PEC Landscape dataset and the KPMP Human Kidney Tissue Atlas dataset (Table 1) [9, 12], and identified gene transcripts that were positively or negatively correlated with *Aldh1a2* mRNA expression levels in scRNA-seq analyses of all mouse PECs and PEC-A1, A2, A3, A4 and PEC-B, separately (Supplementary Table S10) and those correlated with *ALDH1A2* mRNA expression in both scRNA-seq and snRNA-seq analyses of all human PECs, as well as PECs of healthy subjects and patients with AKI or CKD (Supplementary Tables S11). As shown in Figures 5A, B, *Isyna1* (r = 0.51) and *Cystm1* (r = −0.33) are the top genes positively and negatively correlated with *Aldh1a2* in mouse PECs, respectively. Notably, both genes are highly differentially expressed in mouse PEC-A and PEC-B subtypes. An analysis of the human PEC scRNA-seq dataset revealed *SEMA3D* and *COLA1A1* as the most correlated with *ALDH1A2*, but both have low correlation coefficients (r = 0.19 and −0.12, respectively, Figures 5C, D). In snRNA-seq analysis of human PECs, *RBFOX1* (r = 0.35) and *ROBO2* (r = −0.24) are the most significantly positively and negatively correlated with *ALDH1A2*, respectively (Figures 5E, F). Interestingly, both *ALDH1A2* and *RBFOX1* are among the top-3 most specifically expressed genes in human PECs (Supplementary Table S4), while *ROBO2* expression in the AKI dataset appears different from healthy control and CKD dataset, i.e., PECs with high *ALDH1A2* and repressed *ROBO2* expression are enriched in AKI (Figure 5F).

**FIGURE 6:**
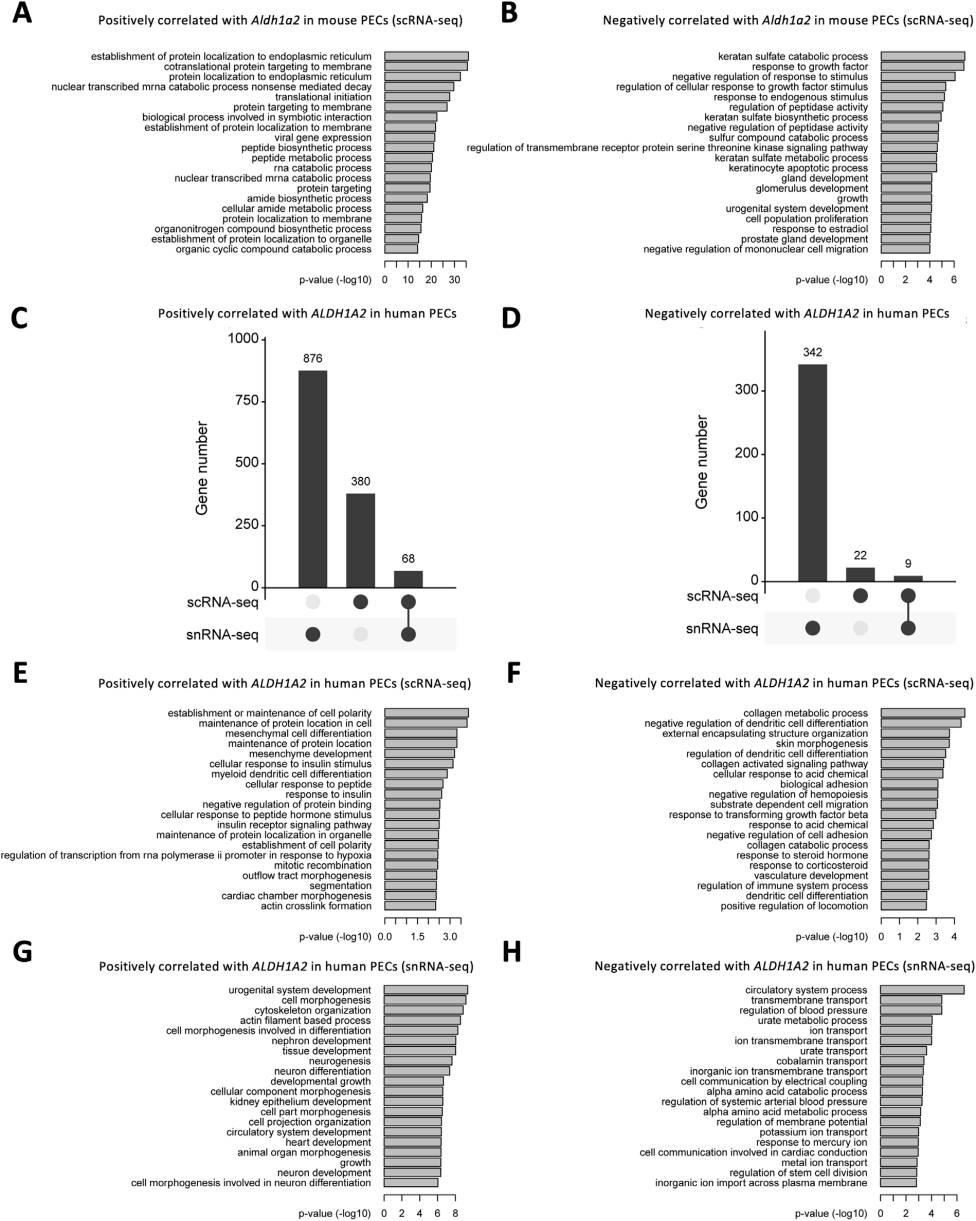
Bioinformatic analyses of genes correlated with *Aldh1a2/ALDH1A2* expression in mouse and human PECs. **(A,B)** Bar plots showing the -log10 (P-value) of enrichment analysis of representative Gene Ontology biological pathways among the gene list with positive **(A)** and negative **(B)** correlations with *Aldh1a2* in all mouse PECs of the Mouse PEC Landscape scRNA-seq dataset. **(C,D)** Upset plots showing the distribution of genes with positive **(C)** and negative **(D)** correlations with *ALDH1A2* in the scRNA-seq and snRNA-seq datasets of the KPMP Human Kidney Tissue Atlas. **(E,F)** Bar plots showing the -log10 (P-value) of enrichment analysis of representative Gene Ontology biological pathways among the gene list with positive **(E)** and negative **(F)** correlations with *ALDH1A2* in human PECs in the scRNA-seq dataset of the KPMP Human Kidney Tissue Atlas. **(G,H)** Bar plots showing the -log10 (P-value) of enrichment analysis of representative Gene Ontology biological pathways among the gene list with positive **(G)** and negative **(H)** correlations with *ALDH1A2* in human PECs of the snRNA-seq dataset of the KPMP Human Kidney Tissue Atlas.

We further explored the Gene Ontology pathways enriched in *Aldh1a2*-correlated genes in the mouse scRNA-seq PEC dataset and *ALDH1A2*-correlated genes in scRNA-seq and snRNA-seq human PEC datasets. In mouse PECs, “protein localization to endoplasmic reticulum” and “cotranslational protein targeting to membrane” are the leading pathways enriched by genes positively correlated with *Aldh1a2* (Figure 6A), while enriched pathways among genes negatively correlated with *Aldh1a2* are “keratan sulfate catabolic process,” response to growth factor” and “negative regulation of response to stimuli”, etc (Figure 6B). As shown in Figures 6C, D, genes correlated with *ALDH1A2* in human PECs in the scRNA-seq dataset and those in the snRNA-seq dataset poorly overlap—only 5.1% and 2.4% of the genes positively and negatively correlated with *ALDH1A2* are shared in the two datasets, respectively. Hence, unsurprisingly, Gene Ontology pathways enriched among those genes correlated with *ALDH1A2* in human PECs annotated in the scRNA-seq and snRNA-seq datasets also vary. While “establishment or maintenance of cell polarity” and “maintenance of protein location in cell” are the top-2 pathways enriched by genes positively correlated with *ALDH1A2* in the scRNA-seq dataset, those most enriched in the snRNA-seq dataset are “urogenital system development” and “cell morphogenesis” (Figures 6E, G). The top-2 pathways enriched by genes negatively correlated with *ALDH1A2* in the scRNA-seq dataset are “collagen metabolic process” and “negative regulation of dendritic cell differentiation,” while the top-2 pathways enriched by genes negatively correlated with *ALDH1A2* in the snRNA-seq dataset are “circulatory system process” and “transmembrane transport” (Figures 6F, H). It awaits further investigation to answer why scRNA-seq and snRNA-seq datasets give rise to largely different sets of *ALDH1A2*-correlated genes and their related Gene Ontology pathways and to understand the biological implications of these findings.

**FIGURE 7:**
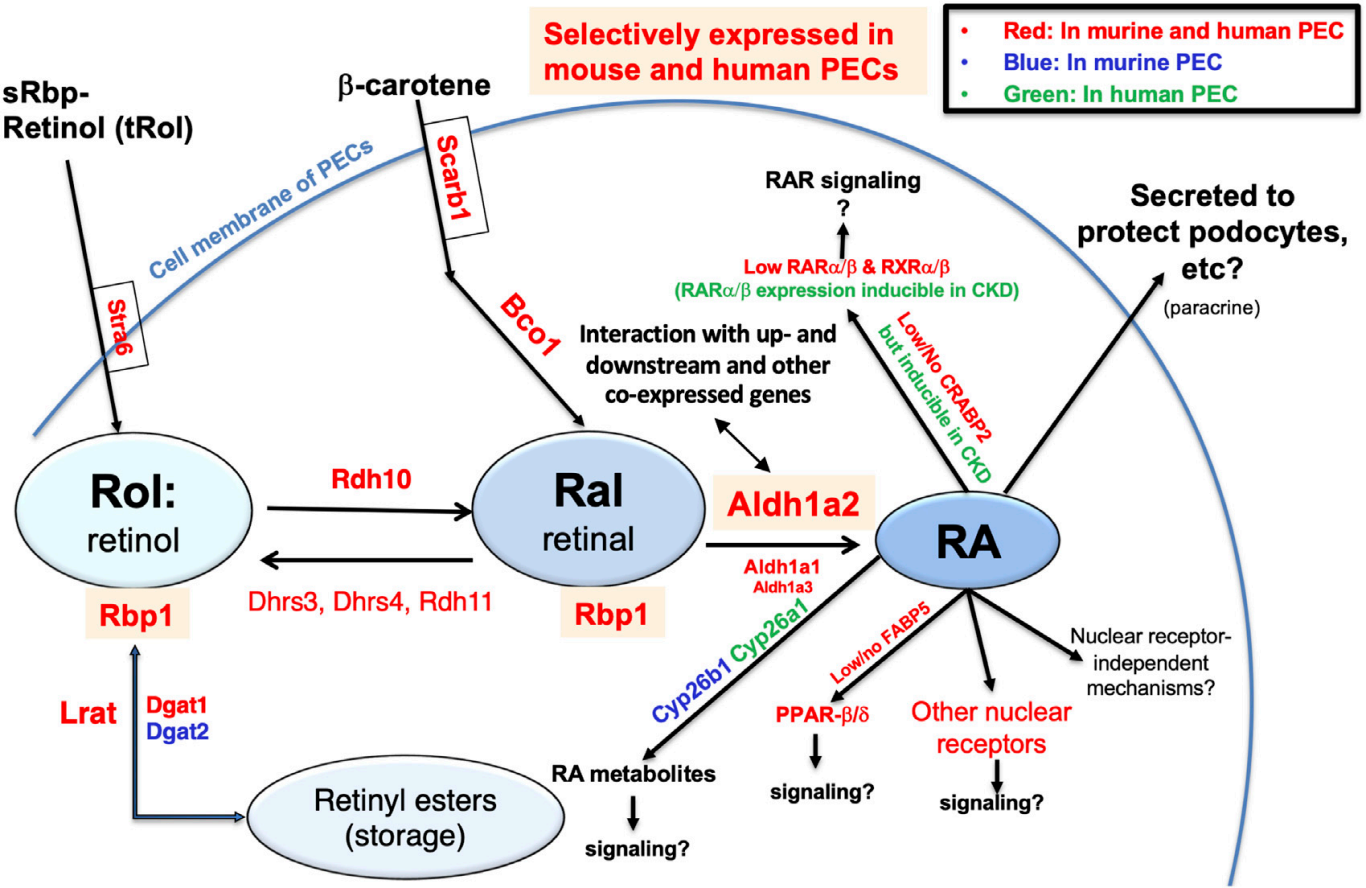
Retinol metabolon and RA signaling in human and mouse PECs as inferred by physiological (and pathophysiological) mRNA expression levels in scRNA-seq and snRNA-seq databases. Mouse PEC gene expression data extracted from Kidney Cell Explorer (Table 1) and human PEC gene expression data extracted from the Susztak Lab Kidney Biobank (Table 1) were used to predict the expression of proteins in the retinol metabolon and RA signaling pathways in mouse and human PECs. These included proteins involved in cellular uptake of retinol and β-carotene; enzymes catalyzing conversion among retinol, retinyl esters and retinaldehyde (retinal); RA synthesizing and catabolizing enzymes; intracellular carrier proteins of retinoids; and receptors and signaling molecules mediating the biological effects of RA. Components expressed in PECs of mice, humans, and both species, as predicted by scRNA-seq and snRNA-seq data, are encoded by blue, green and red fonts, respectively. Retinyl esters, retinol, retinal, RA, and proteins selectively expressed in both mouse and human PECs are highlighted by color shades.

Finally, to generate an integrated understanding of the expression and role of the *Aldh1a2*/*ALDH1A2* genes in mouse and human PECs, against the backdrop of the combined retinol metabolon and RA signaling pathway, we used the Kidney Cell Explorer mouse kidney scRNA-seq data and the Susztak Lab human kidney snRNA-seq data (Table 1) to predict the retinol metabolon and RA signaling in PECs of both species. In brief, as summarized in Figure 7, both mouse and human PECs are predicted to express receptors mediating cellular retinol and β-carotene uptake, carrier proteins mediating intracellular retinol and retinaldehyde transport, enzymes catalyzing interconversion between retinol, retinyl esters and retinaldehyde, as well as enzymes involved in RA synthesis and catabolism. These predictions, if confirmed experimentally, suggest that *Aldh1a2*/*ALDH1A2* may exert their functions in PECs through RA-mediated signaling pathways. In PECs of both species, the findings of low or no expression of *Crabp2/CRABP2* and *Fabp5/FABP5*, low expression of *Ppard/PPARD* and RAR and RXR isotypes, and high expression of other nuclear receptors that heterodimerize with RXRs neither support nor exclude the possibility of RA-RAR or RA-*PPARβ/δ* signaling in PECs. Other nuclear receptor-dependent and independent signaling induced by RA or RA metabolites could also have a role to play. Additionally, RA release could act on other cells, including podocytes, through a paracrine mechanism. In CKD patients, *CRABP2*, *RARA* and *RARB* expression in PECs were induced [15], suggesting that physiological and pathological RA signaling pathways might differ.

## Discussion

Recent scRNA-seq studies have shown that *Aldh1a2* mRNA expression in PECs changes differentially in different mouse glomerular disease models, ranging from a substantial increase in anti-GBM glomerulonephritis (Figures 2C, F), marked repression in CKD (Figure 3A), and moderate induction in mice undergoing ischemia-reperfusion-induced AKI (Figure 3D), to a moderate repression in mouse models of FSGS manifesting nephrotic syndrome (Supplementary Figure S1). ScRNA-seq and snRNA-seq clinical studies also show diminished *ALDH1A2* mRNA expression in PECs in patients with CKD, including those with diabetes, hypertension and ADPKD. So far, scRNA-seq or snRNA-seq studies directly comparing kidney tissues from patients with anti-GBM glomerulonephritis, MCD and FSGS have not yet been reported. Given the aforementioned findings, further clinical studies on the genetics, epigenetics and gene expression of *ALDH1A2* and genes encoding other components of the retinol metabolon and RA signaling in healthy subjects and patients with kidney disease are warranted.

Consistent with scRNA-seq and snRNA-seq-based findings, human PECs have been reported to manifest ALDH1A activity that is repressed by an ALDH1A inhibitor [21] and ALDH1A2 shows positive staining in PECs in normal human kidneys, as documented by the Human Protein Atlas (Table 1). PECs have been reported to stain positive with an *Aldh1a1/2* antibody in both healthy and FSGS mice, also supporting the observation that Aldh1a2 protein is expressed in mouse PECs [21]. Using *RARE-LacZ* mice as a reporter of RA-RAR-dependent transcriptional activity, PECs do not show any physiological RA-RAR activity. However, this activity can be triggered upon podocyte injury 4 days after doxorubicin-induced podocyte injury and this activity is subsequently switched off as proteinuria becomes heavier, due to albumin sequestration of RA and hence preventing RA from activating RAR [21].

In young mice and in cultured PECs, activation of RA/RAR signaling induces PEC differentiation into podocytes, while inhibition of this signaling impairs the regeneration of podocytes. In contrast, RA rescues podocyte regeneration repressed by heavy albuminuria and ameliorates FSGS [21]. In view that RA signaling through RARs triggers PEC differentiation into podocytes, it makes sense that RA signaling via RAR is physiologically inhibited to preserve the PEC phenotype. In FSGS mice, most PECs showing RA/RAR activity are outside FSGS lesions while most of those within FSGS lesions do not respond to RA and do not differentiate into podocytes, suggesting that in addition to albuminuria, other local molecular and cellular factors in the FSGS niche may also affect RA/RAR signaling in the PECs [21].

Mechanisms remain elusive for how RAR signaling in PECs is physiologically repressed, how this signaling is activated upon podocyte injury, and whether these pathophysiological processes observed in FSGS mouse models are also at play in humans. These are important questions to answer and the related mechanistic insights could be harnessed for the treatment of FSGS.

The substantial induction of *Aldh1a2* mRNA expression in PECs of anti-GBM glomerulonephritis mice (Figures 2C, F) and the substantial repression of *Aldh1a2* mRNA expression in PECs in a murine model of CKD (Figure 3A) are important findings. Given the well-established hormetic effects of RA [7], these changes in *Aldh1a2* expression and subsequent RA activity could have profound effects on PECs and their crosstalk with other cells. To determine the role of *Aldh1a2* in PECs, conditional, PEC-selective *Aldh1a2* knockout, RNA silencing and/or strategies for rescuing repressed Aldh1a2 activity in PECs, *e.g.,* PEC-selective *Aldh1a2* transgenic mice, could be useful tools. *Aldh1a* antisense oligonucleotides and other types of Aldh1a inhibitors, RA inactivation inhibitors, as well as RAR and RXR agonists and antagonists could also be useful tools in experimental studies.

Dysregulation of retinol metabolism and RA signaling in type-1 and type-2 diabetes and diabetic nephropathy and the efficacy of RA in treating experimental models of diabetic nephropathy are all well documented [22–27]. In particular, proteomic analysis of renal cortices of wild-type control and *db/db* mice has identified RA as a key dysregulated signaling hub in type-2 diabetes, characterized by dysregulated RA-synthesizing enzymes and reduced RA biosynthesis [23]. In *db/db* mice and *ob/ob* mice (loss-of-function mutation of the leptin gene), O-GlcNAcylation, a posttranslational modification that adds O-linked β-N-acetylglucosamine to serine or threonine residues of many proteins including Stra6/STRA6 and Aldh1a1/ALDH1A1, is significantly increased in diabetic kidney tissues [24]. This leads to suppressed retinol metabolism and downstream signaling [24]. RNA-seq analysis of kidney biopsy samples from patients with early and advanced diabetic nephropathy and of normal kidney tissues identified biphasic changes in expression of genes involved in the RA pathway, characterized by an upregulation in the early stage, but downregulation in the late stage, of diabetic nephropathy [25]. These results, together with the scRNA-seq and snRNA-seq data from diabetic nephropathy murine models and patients (Figures 3A–C, 4C) suggest the need for better understanding of the dynamics of retinol metabolon and RA signaling in different stages of diabetes and diabetic nephropathy, and the importance of further investigations at translational, posttranslational and bioactivity levels.

Few studies referenced in this report have directly examined the effects of sex on the expression and activity of the retinol metabolon and RA signaling in the kidney. The only exception is the Mouse Kidney Cell Explorer database, in which sex-specific differences in gene expression in kidney proximal tubules have been well documented, including the female selective expression of *Stra6* in segment 1 proximal tubule cells (Figure 2B) [8]. Another hint of possible sex differences in *Aldh1a2* mRNA expression is observed in two mouse studies from the same laboratory, one using males only, while the other using females only (Figures 3A, D). However, without direct comparison in well-designed studies one cannot be sure that the different *Aldh1a2* mRNA expressing percentages in these reports are sex-dependent. Future studies should directly compare both sexes.

Mechanistically, many important questions await answers. Although likely, whether effects of Aldh1a2/ALDH1A2 in PECs are mediated by RA synthesis remains unknown. If so, which RA subtypes and signaling pathways are operative in PECs in health and disease? Given that *Aldh1a2* is differentially expressed in PEC subtypes, which may interconvert, it is important to identify the role of *Aldh1a2* in the interconversion among PEC subtypes. For example, as platelet-derived growth factor receptor (PDGFR) signaling plays a role in FSGS [28], and *Pdgfra and Pdgfrb* expression are both strongly expressed in PEC-B but not in PEC-A isotypes [9], it will be intriguing to learn whether changes in *Aldh1a2* expression and RA signaling play any role in the increased PEC-B cell numbers in anti-GBM glomerulonephritis, FSGS, and other kidney diseases [9, 28] and whether and how Aldh1a2, RA and PDGFR signaling pathways crosstalk in PECs. As some PECs are progenitor cells [21, 29], it will be important to determine the roles of *Aldh1a2* expression and RA signaling in the stemness of these progenitor subpopulations and in their trans-differentiation into podocytes and proximal tubular cells [21, 29]. Finally, repression of *Aldh1a2*/ALDH1A2 in PECs in both a murine CKD model (Figure 3A) and in patients with CKD (Figure 4C) is in stark contrast with enhanced *Aldh1a2* expression in PECs and fibroblasts in mice with AKI induced by ischemia-reperfusion, peaking at 12 h and 2 days, and remaining elevated 6 weeks after ischemia-reperfusion, when kidney functions have returned to normal [11]. The authors did not observe these mice beyond 6 weeks. Future research will need to examine AKI models of different etiologies and severity and follow up longer to examine whether different types and severity of AKI have different *Aldh1a2* responses and whether AKI-to-CKD transition is accompanied with repressed *Aldh1a2* expression, and if so, whether such changes in *Aldh1a2* expression play a causal role in AKI-to-CKD transition.

In conclusion, snRNA-seq and scRNA-seq studies have identified PECs as the major site of expression of the *Aldh1a2/ALDH1A2* gene. Current evidence has led us to hypothesize that RA biosynthesis catalyzed by Aldh1a2/ALDH1A2 in PECs may play an important role in kidney health and disease in an autocrine and/or paracrine fashion. We have previously reported that RA/RAR activity is physiologically confined to the collecting duct in mouse kidneys [19, 30] and that in cultured collecting duct cells, several genes implicated in the defense against kidney injury are targets of the endogenous RA/RAR activity [31], and that the RA/RAR activity in collecting duct cells is regulated differentially by AKI and CKD mediators [19]. We have further hypothesized a major defense role for the RA/RAR signaling in the collecting duct, especially PCs, in renal tubulointerstitial injury [32].

Taken together, research addressing the roles of Aldh1a2/ALDH1A2 in PECs and of RA/RAR activity in collecting duct cells promises to shed new light on the mechanisms of glomerular and tubulointerstitial defense, how this defense is overcome in disease, and how properly restoring dysregulated defense could be developed into novel therapies. In particular, harnessing insights from these two lines of research, retinoid and non-retinoid therapies regulating RAR-dependent and RAR-independent RA signaling pathways could be developed to maximize the beneficial effects while minimizing unwanted effects of retinoids [33, 34].
