# Supplementary material for: Single-cell RNA sequencing data locate ALDH1A2-mediated retinoic acid synthetic pathway to glomerular parietal epithelial cells
Source: Exp Biol Med (Maywood). 2024 Sep 18;249:10167. doi: 10.3389/ebm.2024.10167 (PMC11444976; doi:10.3389/ebm.2024.10167)
Supplement: Supplementary file 2 [file DataSheet1.PDF]

Single-cell RNA sequencing data locate ALDH1A2-mediated retinoic acid synthetic pathway to glomerular parietal epithelial cells

Wen-Bin Liu[a], Damian Fermin[b], An-Long Xu[a,*], Jeffrey B Kopp[c,*], and Qihe Xu[d,*]

[a] Beijing Research Institute of Chinese Medicine, Beijing University of Chinese Medicine, Beijing, China.

[b] Department of Internal Medicine, Division of Nephrology, University of Michigan, Ann Arbor, Michigan, USA.

[c] Kidney Disease Section, Kidney Diseases Branch, NIDDK, NIH, Bethesda, Maryland, USA.

[d] Renal Sciences and Integrative Chinese Medicine Laboratory, Department of Inflammation Biology, School of Immunology & Microbial Sciences, Faculty of Life Sciences & Medicine, King's College London, UK.

Supplementary materials

Materials	Page No.
Suppl. fig. 1	1
Suppl. table 1	2
Suppl. table 2	2
Suppl. table 3	3-5
Suppl. table 4	6
Suppl. table 5	7
Suppl. table 6	7
Suppl. table 7	7
Suppl. table 8	8
Suppl. table 9	8

Suppl. fig. 1

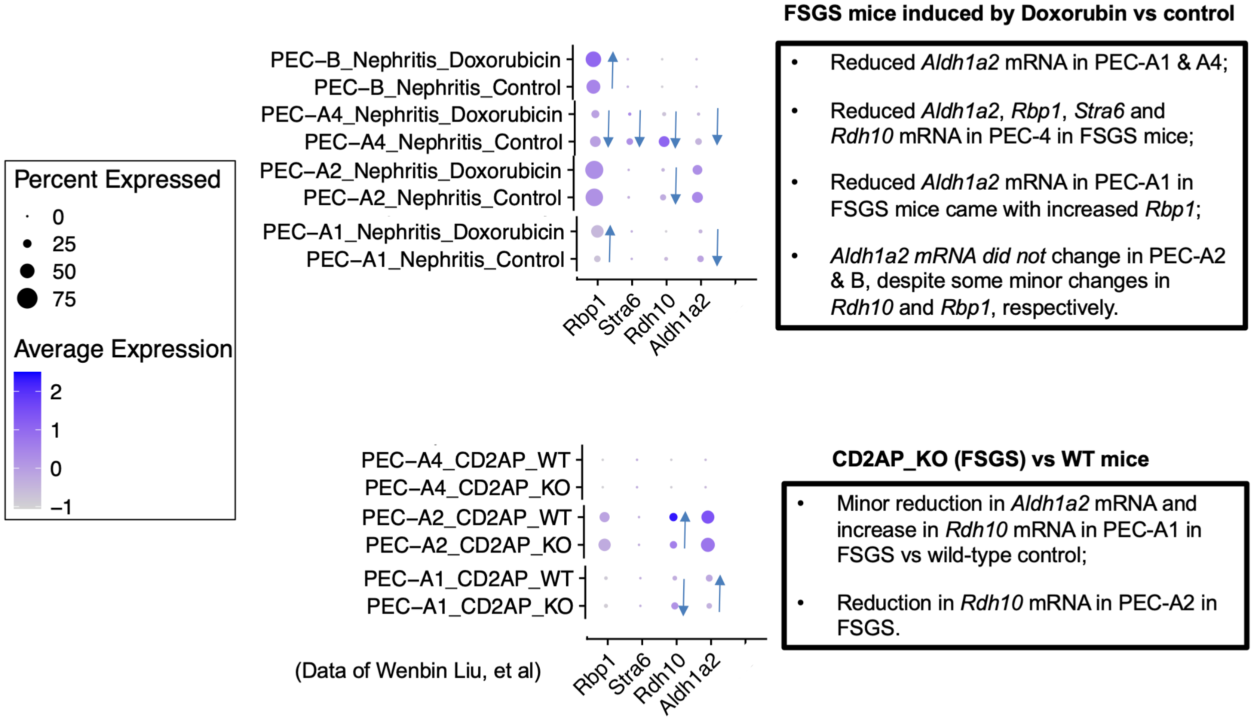

Suppl. fig. 1. scRNA-seq analysis of healthy control versus FSGS mice induced by doxorubicin or *Cd2ap* gene knockout revealed no increase in *Aldh1a2* mRNA expression in any PEC subtypes, and possibly lower expression in some PEC subtypes in FSGS mice. Dot sizes indicate the proportion of PECs expressing a specific gene; brighter blue color indicates higher expression levels. The trend of changes in expression is indicated by blue arrows pointing to the higher expression. FSGS: focal segmental glomerulosclerosis; WT: wild-type; Cd2ap_KO: *Cd2ap* gene knockout mice; Nephritis_ doxorubicin and Nephritis_ Control: FSGS mouse model induced by doxorubicin and their corresponding healthy control mice, respectively.

Suppl. table 1. scRNA-seq: PECs had highest *ALDH1A2* expression among all renal cell types in healthy humans. Shown are cell types with higher *ALDH1A2* expression than all other cell types combined.

clusterAbbrev	clusterName	cellCount	meanExp	pctCellsExpressing	foldChange	pVal	pValAdj
**C-TAL**	Cortical Thick Ascending Limb Cell	3471	0.447	27.9	0.454	4.73E-95	1.44E-90
**PEC**	Parietal Epithelial Cell	158	1.01	34.8	0.853	1.26E-32	3.84E-28
**dPT/DTL**	Proximal Tubule Epithelial Cell / Descending Thin Limb Cell (*degenerative*[3])	214	0.639	22.9	0.557	2.19E-17	6.66E-13
**M-TAL**	Medullary Thick Ascending Limb Cell	41	0.622	22.0	0.536	0.000606	1.00

[3] *degenerative*: Marked loss of differentiation markers, and/or increased percentages of endoplasmic reticulum stress transcripts (% ERT) and mitochondrial transcripts (%MT) and/or marked decrease in genes detected. These cells could represent an early injury state or cells that will not recover function. ADJ P Value =1.00: adjusted p-values showing values of 1 means inability to reject the null hypothesis regardless of the P VALUE.

The results are based on data generated by the Kidney Precision Medicine Project. Accessed on 6th February 2024. https://atlas.kpmp.org/explorer/dataviz. Funded by the National Institute of Diabetes and Digestive and Kidney Diseases (Grant numbers: U01DK133081, U01DK133091, U01DK133092, U01DK133093, U01DK133095, U01DK133097, U01DK114866, U01DK114908, U01DK133090, U01DK133113, U01DK133766, U01DK133768, U01DK114907, U01DK114920, U01DK114923, U01DK114933, U24DK114886, UH3DK114926, UH3DK114861, UH3DK114915, UH3DK114937)

Suppl. table 2. snRNA-seq: PECs had the highest *ALDH1A2* expression among all renal cell types in healthy humans**.** Shown are cell types with higher *ALDH1A2* expression than all other cells combined. The source of data is the same as in Suppl. table 1.

clusterAbbrev	clusterName	cellCount	meanExp	pctCellsExpressing	foldChange	pVal	pValAdj
**PEC**	Parietal Epithelial Cell	527	40.4	88.2	4.14	2.17E-251	6.45E-247
**DTL1**	Descending Thin Limb Cell Type 1	1524	13.2	63.8	2.58	1.72E-185	5.11E-181
**DTL2**	Descending Thin Limb Cell Type 2	901	5.75	56.4	1.41	1.03E-84	3.07E-80
**M-FIB**	Medullary Fibroblast	4587	3.06	47.2	0.703	1.54E-78	4.58E-74
**aTAL1**	Thick Ascending Limb Cell Cluster 1 (*adaptive / maladaptive / repairing*[1])	2513	2.78	49.9	0.571	1.64E-75	4.87E-71
**PapE**	Papillary Tip Epithelial Cell	322	2.84	58.4	0.577	3.86E-39	1.15E-34
**dDTL3**	Descending Thin Limb Cell Type 3 (*degenerative*[3])	399	3.62	54.9	0.845	1.6E-38	4.76E-34
**M-TAL**	Medullary Thick Ascending Limb Cell	4160	2.43	41.1	0.433	6.77E-36	2.01E-31
**C-TAL**	Cortical Thick Ascending Limb Cell	4205	2.17	39.6	0.315	4E-31	1.19E-26
**MC**	Mesangial Cell	200	3.86	45.5	0.917	6.36E-16	1.89E-11
**aTAL2**	Thick Ascending Limb Cell Cluster 2 (*adaptive / maladaptive / repairing*[1])	194	2.10	44.8	0.264	2.03E-10	0.00000604

[1] *adaptive/maladaptive/repairing*: Represented by cells that retain differentiation markers of reference states, albeit at lower levels, but also show expression of known injury-associated genes, mesenchymal markers or factors promoting inflammation or fibrosis. [3] *degenerative*: as defined in Suppl. table 1.

Suppl. table 3. scRNA-seq: Top 140 mRNAs differentially expressed in human PECs. Based on fold-change vs all other cell types, *ALDH1A2* mRNA is the 129th most differentially expressed marker of PECs. The source of data is the same as in Suppl. table 1.

gene	foldChange	pVal	pValAdj
**CTGF**	3.91	3.56E-306	1.08E-301
**CFH**	3.38	0	0
**PTGDS**	2.78	5.79E-240	1.76E-235
**VCAM1**	2.77	2.15E-278	6.55E-274
**BGN**	2.37	4.8E-254	1.46E-249
**CLDN1**	2.23	1.43E-290	4.35E-286
**TNNT2**	2.17	3.12E-245	9.49E-241
**IGFBP7**	2.11	7.14E-185	2.17E-180
**CYP1B1**	1.99	4.53E-162	1.38E-157
**IGFBP2**	1.93	2.75E-136	8.35E-132
**MGP**	1.93	4.66E-164	1.42E-159
**BCAM**	1.92	1.11E-140	3.37E-136
**TPM1**	1.90	5.5E-153	1.67E-148
**TIMP1**	1.87	2.42E-130	7.35E-126
**SLC4A11**	1.84	5.36E-189	1.63E-184
**CTSC**	1.81	1.17E-110	3.56E-106
**IFITM3**	1.70	1.2E-134	3.64E-130
**DAPL1**	1.69	2.12E-150	6.44E-146
**CCL2**	1.69	1.9E-61	5.78E-57
**VIM**	1.69	4.71E-145	1.43E-140
**THY1**	1.68	1.71E-124	5.2E-120
**SPARC**	1.65	2.16E-194	6.56E-190
**CRIM1**	1.63	1.99E-105	6.04E-101
**MMP7**	1.63	1.92E-42	5.85E-38
**SPOCK2**	1.62	1.21E-168	3.69E-164
**C3**	1.62	2.24E-40	6.81E-36
**AEBP1**	1.62	1.7E-189	5.16E-185
**KLK6**	1.61	6.44E-178	1.96E-173
**RIPPLY1**	1.60	4.13E-77	1.25E-72
**NUPR1**	1.56	5.73E-135	1.74E-130
**THBS1**	1.53	4.96E-102	1.51E-97
**SLC48A1**	1.49	2.67E-110	8.12E-106
**CDH6**	1.46	1.66E-113	5.04E-109
**NPR 1.00**	1.44	9.67E-171	2.94E-166
**PDZK1IP1**	1.38	2E-125	6.06E-121
**SLPI**	1.37	1.66E-61	5.05E-57
**SPON2**	1.36	6.45E-116	1.96E-111
**KRT19**	1.35	4.13E-88	1.25E-83
**SYNE1**	1.35	4.99E-93	1.52E-88
**GPC4**	1.35	1.63E-95	4.94E-91
**PALLD**	1.34	1.11E-109	3.37E-105

**MYL9**	1.33	3.25E-135	9.89E-131
**CXCL1**	1.33	1.28E-63	3.9E-59
**SFRP2**	1.33	1.25E-60	3.81E-56
**ANXA1**	1.31	3.49E-121	1.06E-116
**AKAP12**	1.31	2.38E-133	7.25E-129
**ANXA2**	1.29	5.69E-93	1.73E-88
**COL6A2**	1.28	4.02E-152	1.22E-147
**CEBPD**	1.25	2E-44	6.08E-40
**FHL2**	1.25	1.6E-128	4.87E-124
**COL3A1**	1.24	4.91E-91	1.49E-86
**S100A13**	1.22	7.6E-92	2.31E-87
**ARHGAP29**	1.22	2.74E-79	8.34E-75
**RARRES2**	1.21	1.57E-82	4.78E-78
**COL4A2**	1.20	1.41E-94	4.28E-90
**GMDS**	1.20	4.81E-92	1.46E-87
**WT1**	1.19	1.13E-155	3.44E-151
**ERP27**	1.17	2.3E-59	7E-55
**FAT1**	1.17	2.47E-87	7.51E-83
**MXRA8**	1.16	2.85E-135	8.67E-131
**CD151**	1.16	3.24E-83	9.86E-79
**CAV1**	1.15	1.13E-128	3.42E-124
**DCBLD2**	1.14	7.81E-67	2.37E-62
**LGALS1**	1.14	4.52E-40	1.37E-35
**PAWR**	1.14	2.03E-70	6.16E-66
**TNNI1**	1.12	2.19E-121	6.64E-117
**BMP7**	1.11	1.04E-131	3.16E-127
**PRSS23**	1.10	5.98E-59	1.82E-54
**ITM2B**	1.10	3.11E-111	9.45E-107
**LAMP5**	1.09	2.77E-112	8.41E-108
**RERG**	1.08	1.64E-102	4.99E-98
**LY6E**	1.07	1.17E-79	3.55E-75
**COL1A1**	1.06	7.54E-37	2.29E-32
**TNC**	1.06	3.2E-81	9.74E-77
**UGCG**	1.04	1.99E-67	6.04E-63
**TSPAN12**	1.04	1.21E-51	3.67E-47
**RAPGEF3**	1.03	1.93E-62	5.87E-58
**PCP4**	1.03	5.36E-55	1.63E-50
**ITGA3**	1.02	2.06E-63	6.27E-59
**CRB2**	1.02	5.34E-146	1.62E-141
**C1R**	1.02	2.3E-86	6.98E-82
**IGFBP4**	1.02	7.79E-82	2.37E-77
**TNFRSF11B**	1.02	4.43E-57	1.35E-52
**DCDC2**	1.01	7.52E-55	2.29E-50
**ITIH5**	1.01	2.34E-86	7.1E-82
**ITGB8**	1.01	3.25E-61	9.86E-57

**NPR 3.00**	0.998	7.94E-50	2.41E-45
**COL4A1**	0.996	4.61E-64	1.4E-59
**AGRN**	0.992	3.71E-43	1.13E-38
**CTTNBP2**	0.988	1.32E-97	4E-93
**AHNAK**	0.985	2.93E-56	8.9E-52
**TSC22D1**	0.982	7.14E-43	2.17E-38
**TSPAN5**	0.972	2.69E-80	8.17E-76
**EDIL3**	0.964	9.63E-106	2.93E-101
**EVA1B**	0.959	3.32E-60	1.01E-55
**PLA2G16**	0.955	7.21E-58	2.19E-53
**TMEM130**	0.947	4.67E-109	1.42E-104
**NID2**	0.946	2.89E-116	8.77E-112
**CDH2**	0.936	6.85E-84	2.08E-79
**VCAN**	0.930	5.84E-91	1.77E-86
**NR2F2**	0.924	3.47E-52	1.05E-47
**BICC1**	0.921	5.88E-49	1.79E-44
**FOXC1**	0.918	1.33E-83	4.04E-79
**HCFC1R1**	0.907	1.41E-44	4.28E-40
**CAV2**	0.895	9.01E-67	2.74E-62
**STAT1**	0.895	1.88E-46	5.7E-42
**TM4SF1**	0.891	7.09E-114	2.16E-109
**FSTL1**	0.890	1.33E-73	4.05E-69
**KRT17**	0.890	1.36E-62	4.14E-58
**LAPTM4A**	0.882	6.53E-64	1.98E-59
**TNFRSF12A**	0.873	8.38E-30	2.55E-25
**C1S**	0.865	2.36E-68	7.18E-64
**RRAS**	0.857	1.29E-67	3.93E-63
**IL1R1**	0.853	4.64E-45	1.41E-40
**TMEM98**	0.837	4.34E-57	1.32E-52
**EIF3M**	0.834	2.35E-37	7.14E-33
**IFITM2**	0.829	6.71E-46	2.04E-41
**CAVIN3**	0.829	7.13E-63	2.17E-58
**NBL1**	0.823	6.38E-45	1.94E-40
**S100A16**	0.822	9.67E-49	2.94E-44
**FLNA**	0.820	8.58E-41	2.61E-36
**CCND2**	0.811	2.23E-63	6.77E-59
**MT2A**	0.810	5.32E-68	1.62E-63
**SLIT3**	0.806	7.34E-87	2.23E-82
**ADGRG6**	0.800	4.94E-74	1.5E-69
**NRP2**	0.798	4.77E-96	1.45E-91
**ADAMTS1**	0.794	1.04E-44	3.17E-40
**PRKCI**	0.788	1.48E-39	4.49E-35
**ALDH1A2**	0.782	2.02E-70	6.15E-66
**PDE1A**	0.782	2.57E-40	7.82E-36
**CLU**	0.779	1.44E-23	4.38E-19

**EMP3**	0.778	3.56E-50	1.08E-45
**FSTL3**	0.778	3.88E-26	1.18E-21
**SELENOM**	0.769	1.41E-26	4.29E-22
**FN1**	0.769	0.00000166	0.0504
**BHLHE41**	0.769	6.28E-72	1.91E-67
**ARID5B**	0.765	5.92E-35	1.8E-30
**TFF3**	0.764	1.84E-90	5.6E-86
**DPYSL3**	0.759	5.08E-92	1.54E-87
**ANXA5**	0.756	9.04E-38	2.75E-33

Suppl. table 4. snRNA-seq: *ALDH1A2* mRNA is the top differentially expressed marker of PECs; shown are the top 20 mRNAs differentially expressed in PECs. The source of data is the same as in Suppl. table 1.

gene	foldChange	pVal	pValAdj
**ALDH1A2**	3.84	0	0
**LINC01435**	3.53	0	0
**RBFOX1**	3.46	0	0
**FRMD4A**	2.98	4.3E-302	1.28E-297
**CFH**	2.76	4.29E-290	1.28E-285
**KIRREL3**	2.58	5.13E-187	1.53E-182
**NRG3**	2.47	2.13E-213	6.34E-209
**ZFPM2**	2.34	1.12E-253	3.34E-249
**KCNT2**	2.29	4.55E-242	1.35E-237
**PDE1A**	2.21	1.78E-188	5.31E-184
**AC068234.1**	2.20	7.54E-217	2.24E-212
**CTGF**	2.14	7.1E-180	2.11E-175
**SLIT3**	2.06	1.83E-235	5.44E-231
**FAM189A1**	2.04	7.9E-198	2.35E-193
**TSPAN5**	2.03	1.11E-179	3.3E-175
**TENM3**	2.00	5.05E-208	1.5E-203
**FAM155A**	1.95	1.05E-115	3.13E-111
**SYNE1**	1.95	2.05E-257	6.08E-253
**SLC35F4**	1.91	6.36E-133	1.89E-128
**RHEX**	1.90	3.05E-122	9.08E-118

Suppl. table 5. snRNA-seq: *ALDH1A2* expression in healthy control and patients with chronic kidney disease (CKD)

pop	Average Expression (All Samples)	Fraction Expressing (All Samples)	CellType marker p-values	Average Expression (Control Samples)	Fraction Expressing (Control Samples)	Average Expression (Disease Samples)	Fraction Expressing (Disease Samples)	Disease vs. Control p-values
M_TAL	0.79	0.43	0.0e+00	0.93	0.5	0.7	0.4	NS
C_TAL	0.68	0.39	0.0e+00	0.68	0.37	0.65	0.38	5.1e-10
PEC	2.19	0.68	2.1e-143	2.82	0.83	1.8	0.6	2.9e-06
Macula_Densa	0.64	0.38	2.2e-63	0.62	0.36	0.64	0.39	4.5e-02
GS_Stromal	0.26	0.13	2.9e-02	0.09	0.05	0.32	0.16	NS
DCT1	0.57	0.36	3.2e-122	0.56	0.35	0.53	0.34	NS
Ascending_Thin_LOH	0.76	0.45	6.7e-07	NS	NS	0.76	0.45	NS
Mes	0.59	0.29	6.8e-05	0.71	0.35	0.49	0.24	NS
iPT	0.46	0.22	NS	0.43	0.2	0.46	0.22	1.4e-03
B_Naive	0.41	0.18	NS	0.42	0.2	0.37	0.15	NS
Neutrophil	0.39	0.21	NS	0.27	0.19	0.39	0.19	NS
DCT2	0.36	0.22	NS	0.27	0.17	0.44	0.27	1.9e-05
Fibroblast_1	0.36	0.17	NS	0.34	0.15	0.36	0.17	NS
Fibroblast_2	0.35	0.16	NS	0.33	0.16	0.37	0.17	NS
CD4T	0.29	0.15	NS	0.29	0.15	0.28	0.15	NS
NK	0.29	0.23	NS	0.36	0.32	0.22	0.13	NS
CNT	0.27	0.16	NS	0.21	0.12	0.3	0.18	1.9e-05
CD8T	0.27	0.14	NS	0.25	0.14	0.27	0.14	NS
Des-Thin_Limb	0.25	0.13	NS	0.26	0.11	0.24	0.14	NS

Data on PECs are highlighted in red fonts. Extracted from http://www.susztaklab.com/hk_genemap/snRNA.

Suppl. table 6. scRNA-seq: PECs had the highest *ALDH1A2* expression among all renal cell types in CKD patients. Shown are cell types with higher *ALDH1A2* expression than all other cell types combined. The source of data is the same as in Suppl. table 1.

clusterAbbrev	clusterName	cellCount	meanExp	pctCellsExpressing	foldChange	pVal	pValAdj
**aTAL2**	Thick Ascending Limb Cell Cluster 2 (*adaptive / maladaptive / repairing*[1])	3836	0.382	16.9	0.341	8.72E-44	2.65E-39
**PEC**	Parietal Epithelial Cell	268	0.985	27.6	0.841	1.2E-40	3.64E-36
**dDCT**	Distal Convoluted Tubule Cell (*degenerative*[3])	854	0.366	14.2	0.301	1.28E-19	3.9E-15
**MC**	Mesangial Cell	45	1.17	24.4	0.968	1.02E-09	0.0000309

[1] *adaptive/maladaptive/repairing*: The same definition as in Suppl. table 2.

Suppl. table 7. snRNA-seq: PECs had the highest *ALDH1A2* expression among all renal cell types in CKD patients. Shown are cell types with higher *ALDH1A2* expression than all other cell types combined. The source of data is the same as in Suppl. table 1.

clusterAbbrev	clusterName	cellCount	meanExp	pctCellsExpressing	foldChange	pVal	pValAdj
**DTL1**	Descending Thin Limb Cell Type 1	1334	14.2	67.4	2.33	8.68E-182	2.58E-177
**PEC**	Parietal Epithelial Cell	266	26.3	77.1	3.09	4.82E-95	1.43E-90
**DTL2**	Descending Thin Limb Cell Type 2	1255	7.83	58.3	1.47	2.45E-93	7.3E-89
**M-TAL**	Medullary Thick Ascending Limb Cell	3645	4.95	52.9	0.925	3.21E-78	9.55E-74
**M-FIB**	Medullary Fibroblast	1076	6.15	53.6	1.14	1.2E-65	3.57E-61
**C-TAL**	Cortical Thick Ascending Limb Cell	3254	3.22	52.4	0.370	2.87E-61	8.54E-57

Suppl. table 8. scRNA-seq: PECs had the highest *ALDH1A2* expression among all renal cell types in AKI patients. Shown are cell types with higher *ALDH1A2* expression than all other cell types combined. The source of data is the same as in Suppl. table 1.

clusterAbbrev	clusterName	cellCount	meanExp	pctCellsExpressing	foldChange	pVal	pValAdj
**aTAL2**	Thick Ascending Limb Cell Cluster 2 (*adaptive / maladaptive / repairing*[1])	2508	0.336	17.8	0.295	2.35E-50	7.16E-46
**PEC**	Parietal Epithelial Cell	205	0.716	25.4	0.639	1.15E-30	3.49E-26
**aTAL1**	Thick Ascending Limb Cell Cluster 1 (*adaptive / maladaptive / repairing*[1])	764	0.351	11.5	0.296	2.1E-14	6.38E-10
**MC**	Mesangial Cell	43	1.21	23.3	1.00	1.61E-09	0.0000488

[1] *adaptive/maladaptive/repairing*: The same definition as in Suppl. table 2.

Suppl. table 9. snRNA-seq: PECs had the highest *ALDH1A2* expression among all renal cell types in AKI patients. Shown are cell types with higher *ALDH1A2* expression than all other cell types combined. The source of data is the same as in Suppl. table 1.

clusterAbbrev	clusterName	cellCount	meanExp	pctCellsExpressing	foldChange	pVal	pValAdj
**PEC**	Parietal Epithelial Cell	357	38.6	89.4	4.03	1.31E-198	3.89E-194
**DTL1**	Descending Thin Limb Cell Type 1	362	18.9	79.8	2.90	2.09E-146	6.22E-142
**aTAL1**	Thick Ascending Limb Cell Cluster 1 (*adaptive / maladaptive / repairing*[1])	835	3.01	55.9	0.502	4.31E-65	1.28E-60
**C-TAL**	Cortical Thick Ascending Limb Cell	626	3.21	53.5	0.568	2.69E-44	7.99E-40
**DCT1**	Distal Convoluted Tubule Cell Type 1	1715	2.44	46.1	0.280	2.8E-43	8.33E-39
**DTL2**	Descending Thin Limb Cell Type 2	226	5.84	64.2	1.27	2.02E-42	6E-38
**M-FIB**	Medullary Fibroblast	297	4.62	49.8	0.987	5.99E-28	1.78E-23
**aTAL2**	Thick Ascending Limb Cell Cluster 2 (*adaptive / maladaptive / repairing*[1])	175	3.38	52.6	0.619	7.33E-18	2.18E-13
**aFIB**	Fibroblast (*adaptive / maladaptive / repairing*[1])	850	2.49	33.8	0.295	6.95E-11	0.00000207
**DTL3**	Descending Thin Limb Cell Type 3	34	7.71	61.8	1.61	3.75E-09	0.000112
**MC**	Mesangial Cell	76	3.03	40.8	0.494	0.000217	1.00
**ATL**	Ascending Thin Limb Cell	8	3.95	62.5	0.791	0.00445	1.00

[1] *adaptive/maladaptive/repairing*: The same definition as in Table 3. ADJ P Value =1.00: adjusted p-values showing values of 1 means inability to reject the null hypothesis regardless of the P VALUE.
